# Urethrography for diagnostic of penile fracture with ruptured anterior urethra and right corpus cavernosum

**DOI:** 10.1016/j.radcr.2022.08.104

**Published:** 2022-09-30

**Authors:** Purbo Agus Sulistio, Prijambodo

**Affiliations:** Department of Radiology, Faculty of Medicine, Universitas Airlangga, Dr. Soetomo General Hospital, Surabaya, Indonesia

**Keywords:** Penile, Fracture, Rupture, Urethra

## Abstract

Penile fracture is a rare urological emergency that effect due to blunt trauma on erect penis. Mostly often occurs during sexual intercourse and is rare in blunt of trauma from impact at penile. That trauma it can caused by rupture and hematoma of tunica albuginea. In physical examination, we found swelling and hematoma of penile shaft, with abnormal angulation to the right and tenderness. Two hours after sexual intercourse, patient urinated and he felt fresh blood comes out with urine and it is painful. Penile fracture can be diagnostic with clinically and imaging radiology. Important information due clinically with précising radiologist finding can making the correct diagnosis and surgical procedure can be done immediately.

## Introduction

Penile fracture is a rare urological emergency due to trauma to the erect penis, frequently occurring during sexual intercourse, caused by rupture and hematoma of tunica albuginea. The diagnosis is based on physical examination in which patients usually present erected penile with edema. Urethrography fluoroscopy is an excellent diagnostic tool before surgery as it shows the rupture in the cavernous body. We report a case of a 46-year-old man diagnosed with a penile fracture, focusing on the ruptured anterior urethra and right cavernous body.

## Case report

**Fig. 1:**
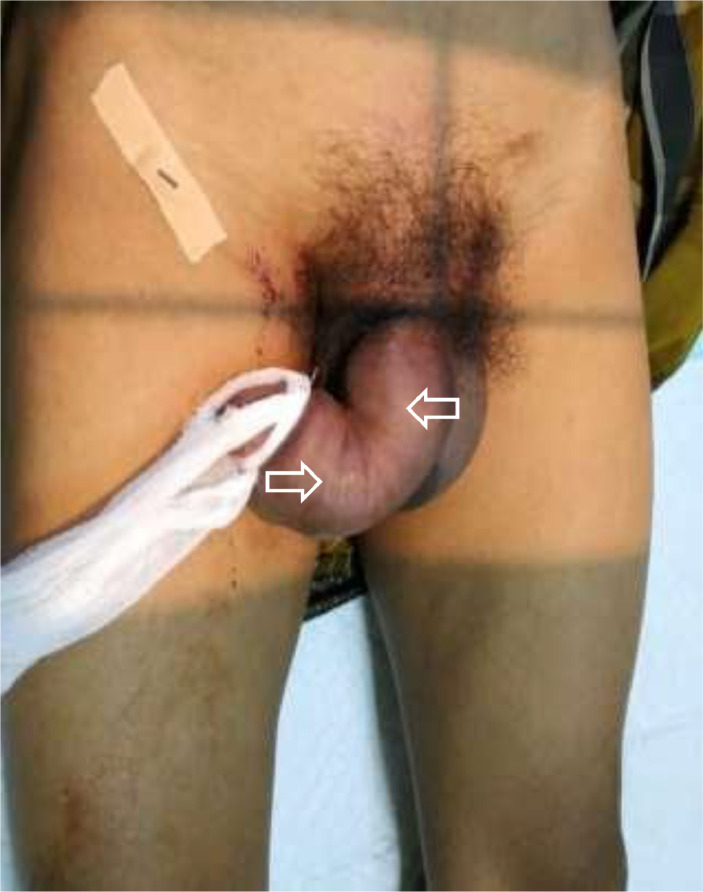
The clinical appearance of the swollen penis with hematoma.

**Fig. 2:**
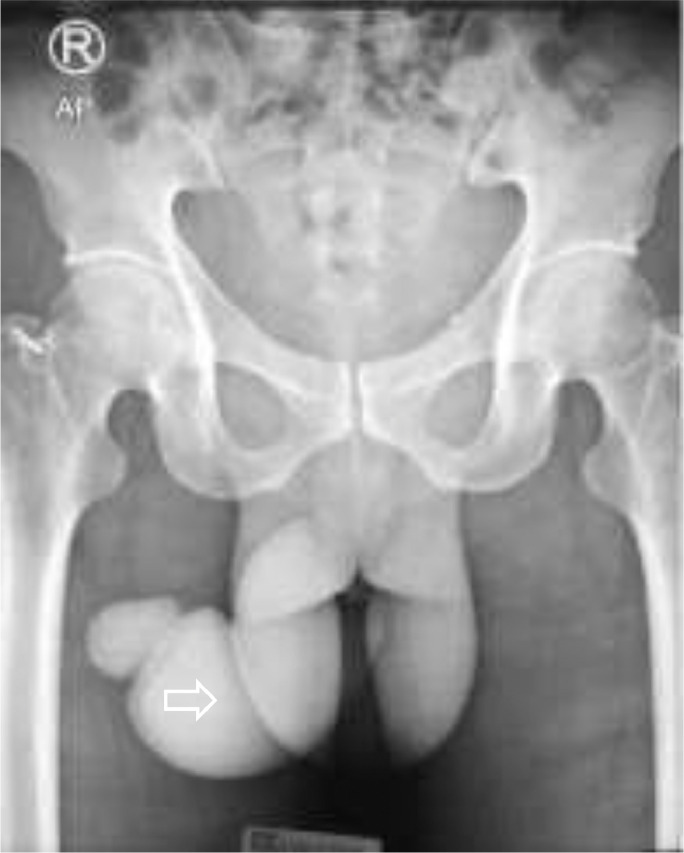
A plain abdominal radiograph showed soft tissue swelling in the penile region, with no diastasis pubic symphysis and opaque shadow in the urinary tract.

**Fig. 3:**
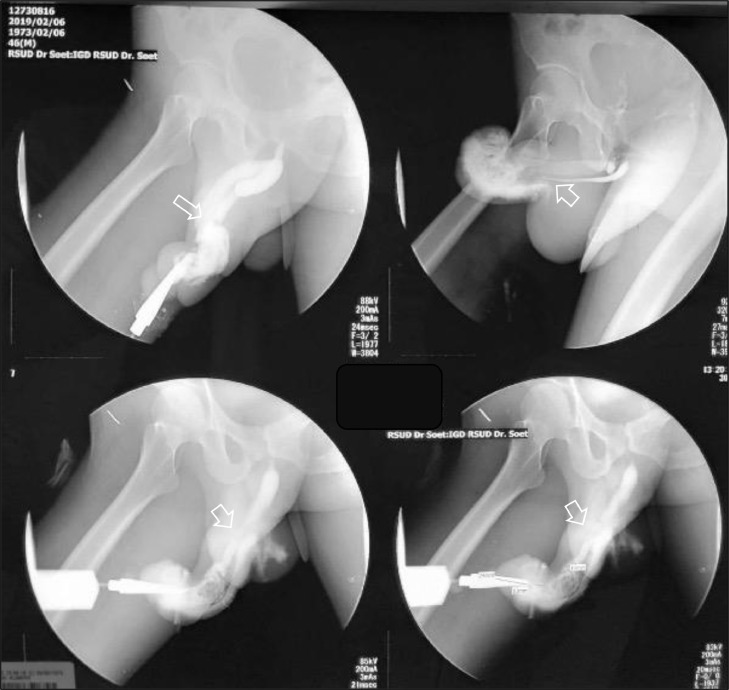
Urethrography with retrograde urethrogram fluoroscopy contrast injected into the penile orifice examination: the contrast leakage, measuring 2.4 cm in length, was evident at around 2.9 cm proximal to the external urethral orifice. The contrast filled the right cavernous body and proximal urethra at the cavernous, bulbous, membranous and prostatic parts, and the bladder. Conclusion: Urethral rupture at the cavernous part at approximately 2.4 cm from the external urethral orifice and ruptured right cavernous body.

A 46-year-old male presented to our emergency unit with pain and edema at the penile. According to his wife, it happened after the sexual intercourse, in which the patient noticed the bloody urine, progressing to swell and darken with pain. The patient's general condition was within normal limits, with a blood pressure of 138/74 mmHg and a pulse of 85 bpm. Localized examination showed the darkened and swollen penile with tenderness (Fig. 1). Laboratory test results were within the normal ranges. An abdominal radiograph and retrograde urethrogram fluoroscopy contrast injected into the penile orifice were conducted afterwards, depicting soft tissue swelling in the penile region (Fig. 2) and a contrast leakage at the distal of the cavernous part of the urethra approximately 2.9 centimeters from the external urethra orifice. We concluded a urethral rupture from these findings (Fig. 3).

**Fig. 4:**
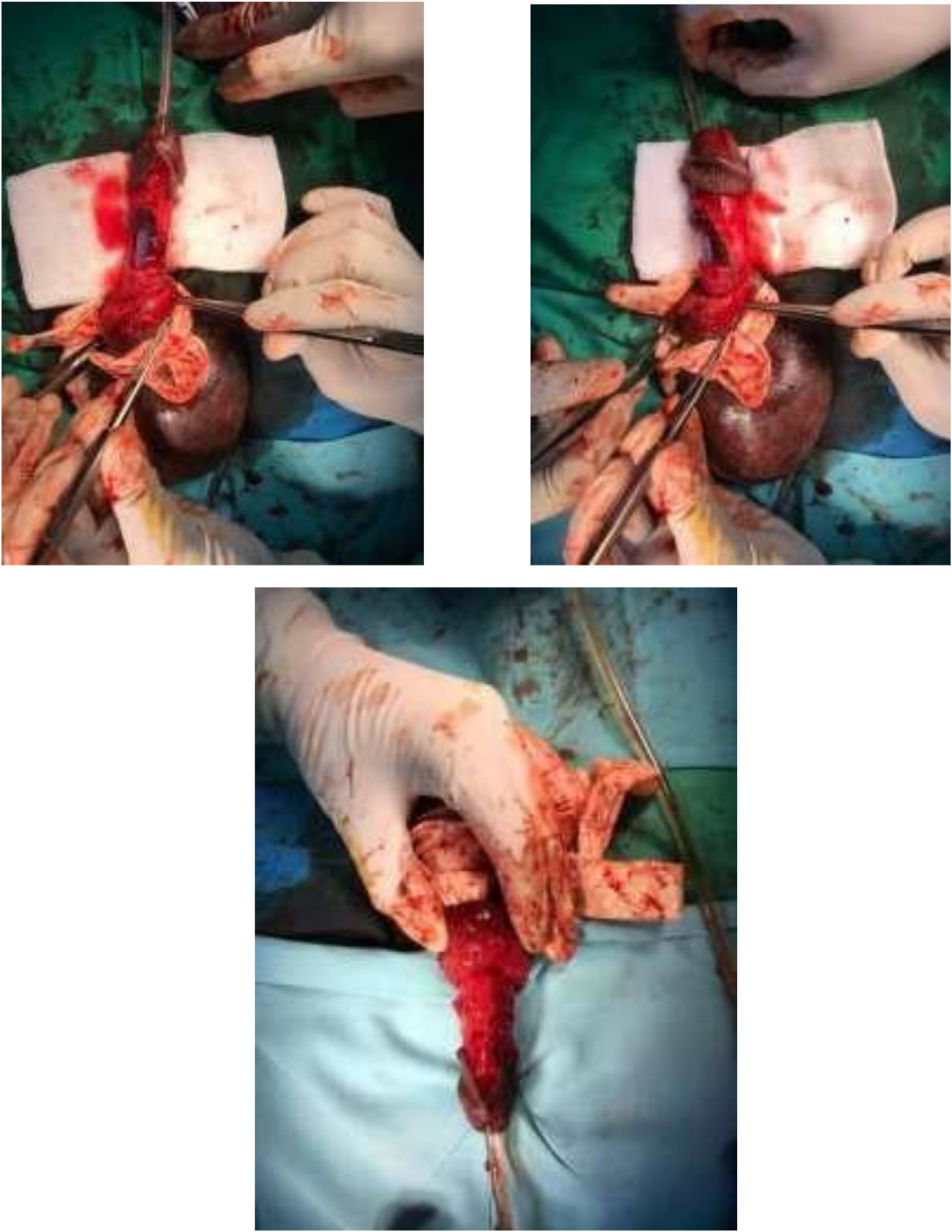
Exploratory procedure at the emergency surgery. A circumferential subcoronal incision and degloving were done to the proximal penis, revealing a hematoma at the dorsolateral side of the right cavernous body which was evacuated afterward, uncovering the ruptured urethra.

**Fig. 5:**
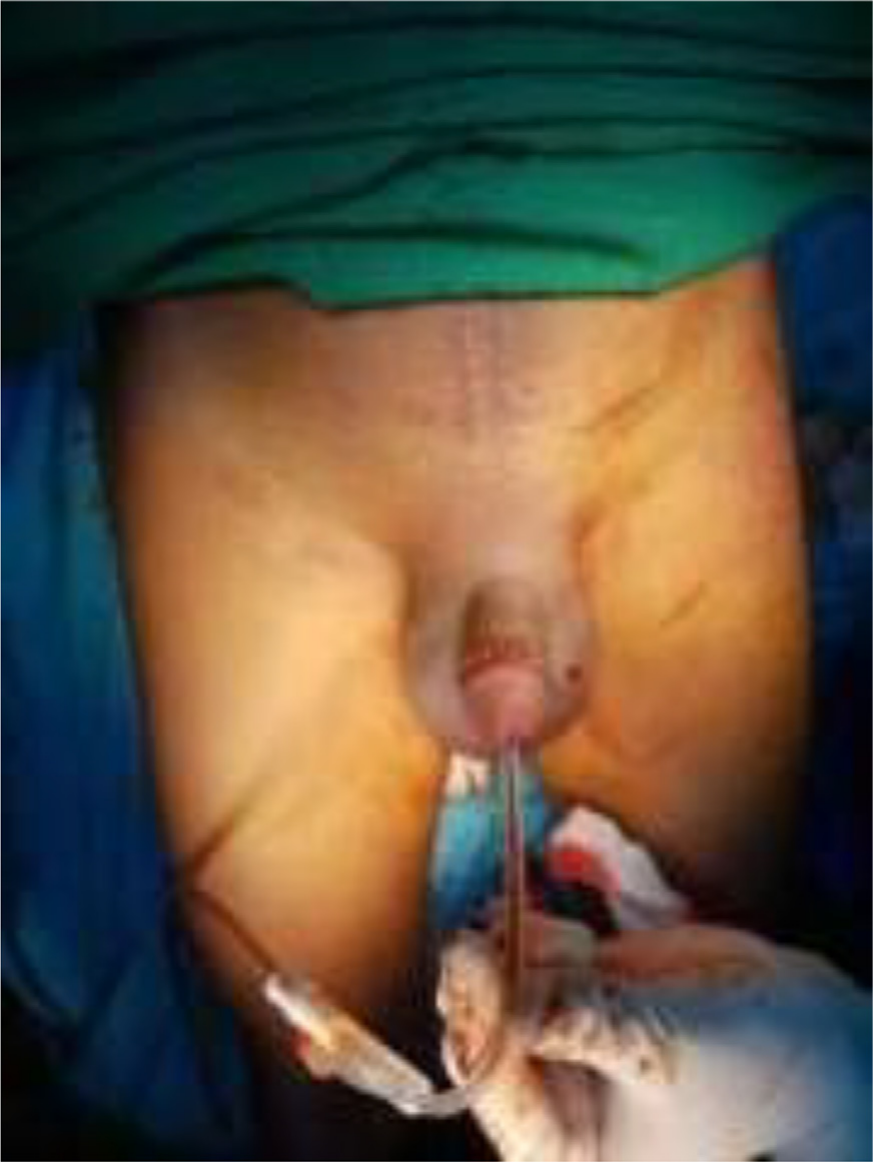
Evacuating the blood clot after surgery; a Foley catheter was inserted to evaluate the urine.

The patient was admitted to the urology surgery department with a diagnosis of a ruptured anterior urethra and right cavernous body and planned for emergency surgical repair as soon as his general condition improved. Exploratory surgery was performed during the repair, with a circumferential subcoronal incision and degloving to the proximal penis, revealing a hematoma on the dorsolateral of the right cavernous body, which was subsequently evacuated. We discovered the ruptured site measuring roughly 1 cm and proceeded to repair it with a primary suture using 3.0 vicryl thread on the torn cavernous body (Fig. 4). The blood clot was removed after the surgery, and a Foley catheter was inserted to evaluate the urine (Fig. 5). There were no intraoperative or postoperative complications, and the patient was discharged within 2 weeks.

**Fig. 6:**
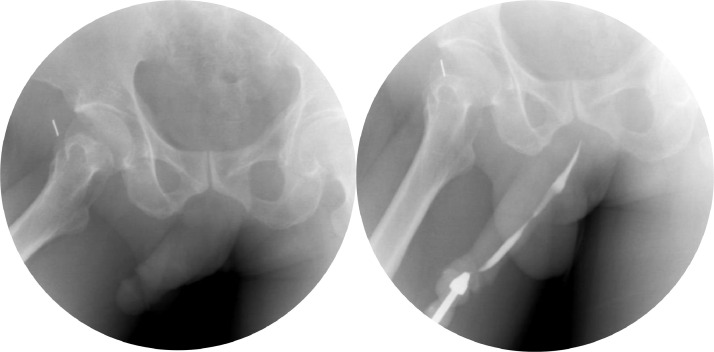
Urethrography with retrograde urethrogram fluoroscopy contrast injected into the penile orifice examination after 2 months of postsurgery showed a stricture at the cavernous part of the urethra, no contrast leakage visualized.

At the 2-month follow-up visit, there was no gross hematuria, swelling, or edema at the penile soft tissue. We requested a contrast urethrography, yielding urethral stricture at the cavernous part with no contrast leakage visualized (Fig. 6).

## Discussion

Erection converts the safe, flaccid penis into a vulnerable organ. During an erection, the thick tunica albuginea becomes thin and fragile. Penile fracture is a relatively rare condition caused by blunt trauma to the erected penis, with the most frequently reported trauma mechanism being non-physiological bending of the erect penis during sexual intercourse or masturbation [1].

Most patients with fractured penis present the symptoms of a cracking sound followed by sudden detumescence with painful penile swelling. The defect at the fractured site is often palpable and has been described as the "rolling sign," referring to a firm, mobile, tender mass where the penile skin can be rolled over the blood clot [1,2]. This classic anamnesis with the local finding of *eggplant* sign of the penis and tenderness at the ruptured site provides the clinical diagnosis of the penile fracture without requisite diagnostic tests in most situations; nonetheless, penile ultrasound, cavernosography, and MRI may be helpful in some cases [2]. Surgical intervention has been shown to have a better outcome and shorter duration of hospitalization with a lower complication rate of erectile dysfunction, penile curvature, and penile pain on erection compared to the conservative treatment. Urethral injury management depends on the extent of the injury. Minimal urethral injuries can be managed with urinary diversion or direct suture of the tear, while the severe cases or complete urethral rupture require spatulated mucosa to mucosa, tension-free anastomosis over urethral catheter [3].

## Conclusion

We reported the case of a penile fracture with ruptured urethra at the cavernous part with retrograde urethrogram fluoroscopy contrast injected into the penile orifice, demonstrated. Penile fracture can be diagnosed with clinical examination and radiology imaging and confirmed upon surgery. Meticulous history taking and precise imaging findings offer a timely diagnosis and prompt surgical repair.

## Patient consent

Written informed consent was obtained from the patient for the publication of this case report. No patient identifiers are disclosed in current report.

## References

[1] Bitsch M, Kromann-Andersen B, Schou J, Sjøntoft E. (1990). The elasticity and the tensile strength of tunica albuginea of the corpora cavernosa. J Urol.

[2] Purnomo BB. (2007).

[3] De-Rose AF, Giglio M, Carmignani G. (2001). Traumatic rupture of the corpora cavernosa: new physiopathologic acquisitions. Urology.

